# Optimization of PCA Error Correction Conditions to Improve Efficiency of Virus Genome De Novo Synthesis

**DOI:** 10.3390/ijms252111514

**Published:** 2024-10-26

**Authors:** Jiazhen Cui, Ao Hu, Xianghua Xiong, Qingyang Wang, Chen Zhu, Zhili Chen, Yuanyuan Lu, Xianzhu Xia, Huipeng Chen, Gang Liu

**Affiliations:** 1 Academy of Military Medical Sciences, Beijing 100850, China; 2 Institutes of Physical Science and Information Technology, Anhui University, Hefei 230000, China

**Keywords:** virus engineering, genome synthesis from scratch, DNA assembly, polymerase chain reaction assembly, splicing efficiency, error correction

## Abstract

In recent years, there have been frequent global outbreaks of viral epidemics such as Zika, COVID-19, and monkeypox, which have had a huge impact on human health and society and have also spurred innovation in virus engineering technology. The rise of synthetic virus genome technology has provided researchers with a new platform to accelerate vaccine and drug development. Although DNA synthesis technology has made significant progress, the current virus genome synthesis technology still requires the assembly of short oligonucleotides of around 60 bp into kb-level lengths when constructing long segments, a process in which the commonly used polymerase chain reaction assembly (PCA) technology has high error rates and is cumbersome to operate. This study optimized the error correction conditions after PCA assembly, increasing the accuracy of synthesizing 1 kb DNA fragments from 4.2 ± 2.1% before error correction to 31.3 ± 3.1% after two rounds of correction, an improvement of over 6 times. This study provides a more efficient operational process for synthesizing virus genomes from scratch, indicating greater potential for virus engineering in epidemic prevention and control and the field of biomedicine.

## 1. Introduction

In recent years, the world has been hit by a series of viral pandemics, including the Zika virus [1,2], the novel coronavirus (SARS-CoV-2) [3,4], and the monkeypox virus [5], which have posed a serious threat to human health and had far-reaching impacts on social development. Virus engineering plays a crucial role in the field of virus prevention and control, providing a solid scientific foundation for effectively preventing the spread of viruses through in-depth research on the basic structure, genetic information, replication mechanism, and other core elements of viruses [6]. Additionally, virus engineering also constructs new vaccine candidates by selectively modifying viruses, such as precisely deleting virulent genes and safely inserting therapeutic genes [7], to safeguard public health security. Today, the synthesis of viral genomes has injected new vitality into the development of virus engineering, providing researchers with a new bottom-up platform to delve into the pathogenesis of viruses and accelerate vaccine development [8,9]. Through synthetic genomics, scientists are able to synthesize viral genomes from scratch, enabling rapid research in this field [10]. This undoubtedly opens up new avenues for virus engineering research and greatly accelerates the research process. For example, in 2021, Pei-Yong Shi et al. ingeniously utilized yeast homologous recombination technology to successfully synthesize the cDNA of SARS-CoV-2 [11]. This major breakthrough provided strong technical support for a deeper understanding of the pathogenic mechanism of SARS-CoV-2 and the development of prevention and control drugs during the COVID-19 pandemic. Furthermore, the rapid development of virus genome synthesis technology has shown remarkable application prospects in various cutting-edge fields such as biopharmaceutical design [12], gene therapy [13,14], and oligonucleotide drug development [15].

Traditional virus engineering is limited to local operations on viruses, such as site-directed mutagenesis. Due to the high specificity and complexity of viruses, the application is somewhat limited. The de novo synthesis of virus genomes greatly complements this technological shortcoming [9]. The currently available DNA synthesis methods are limited in their synthesis capacity and accuracy, making it difficult to directly and accurately synthesize DNA fragments of genome length [16]. Commercial DNA synthesis methods generally rely on assembling shorter kb-level DNA fragments into longer genome DNA. Whether synthesizing virus genomes or other long DNA fragments, it is inevitably necessary to synthesize DNA fragments from oligonucleotides to kb-level lengths [17,18]. Unfortunately, existing technologies cannot directly synthesize kb-level DNA fragments, so how to efficiently and accurately synthesize and assemble these fragments has become a pressing technological challenge.

To address this issue, researchers have explored various synthetic assembly methods, from oligonucleotides to kb-level DNA lengths, including polymerase cycling assembly (PCA) and ligase chain reaction (LCR) [19,20]. In particular, PCA technology, based on the principle of polymerase chain reaction (PCR), can effectively assemble single-stranded oligonucleotides of several tens of bps into double-stranded DNA of several kb, making it one of the most economical and convenient DNA assembly technologies [21]. However, in addition to mismatches caused by improper deprotection during oligonucleotide chemical synthesis in the PCA assembly process, there are also factors such as the damage to the DNA structure by the heat cycling process of enzyme-catalyzed assembly and the fidelity of DNA polymerase used in PCR amplification, which may result in a certain number of base mismatches in the final product [22,23,24]. Although the error rate in the PCA assembly process can be reduced, to some extent, by improving the quality of oligonucleotides and using high-fidelity polymerases, we still need to correct errors more effectively to obtain a higher proportion of correct PCA products. Removing incorrect products from the synthesized gene using specific nucleases, DNA mismatch recognition proteins, etc., is an effective error correction method, such as T7 endonuclease I [25], MutS protein [26,27], etc. T7 endonuclease I distinguishes mismatched products from correct products by catalyzing the cleavage of mismatched sequences with relatively simple operation and wide application, but its efficiency still needs to be further improved.

This study aims to optimize the error correction efficiency of PCA assembly technology by targeting a 1.3 kb DNA fragment. The reason for choosing a target length of 1.3 kb for research is because various errors generated during the DNA fragment synthesis process using PCA technology accumulate as the length of the gene fragment increases, leading to a decrease in the yield of the target product, an increase in synthesis workload, and higher costs. Additionally, recombinant technologies such as Gibson assembly and yeast homologous recombination have difficulty achieving high synthesis efficiency and accuracy at the kb to tens of kb level by adjusting CA assembly conditions and optimizing reaction processes. Assembling DNA fragments of around 1.3 kb is the most cost-effective choice and is more conducive to the efficient progress of our work.

By adjusting the annealing steps in the error correction process from one step to two steps and optimizing the final annealing temperature, we significantly improved the accuracy of the final product. The results show that this improvement greatly increased the correctness of the final product, achieving the same effect with only one round of error correction compared to the original two rounds and also significantly increasing the final accuracy. This research not only provides valuable theoretical guidance and standardized procedures for the rational design and synthesis of kb-level viral genome DNA fragments but also helps to explore the huge potential of viral engineering in the prevention and control of viral epidemics. We look forward to the continuous improvement and optimization of technology, as viral engineering will play an increasingly important role in future epidemic prevention and control and biomedical research.

## 2. Results

### 2.1. Efficiency Analysis of PCA Assembly and One-Step Correction

**Figure 1:**
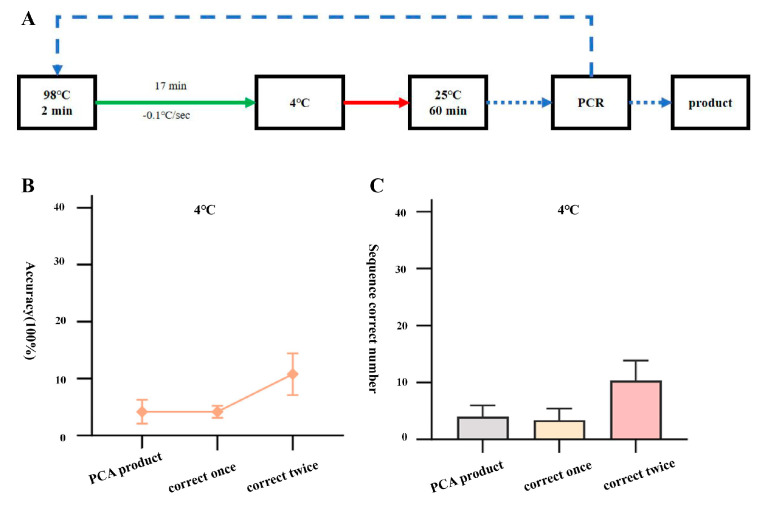
Correction process and efficiency statistics of one-step annealing to 4 °C. (**A**) Flowchart of correction process for one-step annealing to 4°C. (**B**) Line graph showing the accuracy of amplified products after two rounds of correction with PCA assembly products and one-step annealing to 4 °C. (**C**) Number of positive samples of amplified products after two rounds of correction with PCA assembly products and one-step annealing to 4 °C.

We used the high-fidelity polymerase PrimeSTAR HS to assemble three 1.3 kb small DNA fragments (JA, JB, and JC) from the Japanese encephalitis virus vaccine strain (SA14-14-2) by PCA and annealed the assembled products using a one-step error correction at 4 °C. The PCA assembly products were subjected to two rounds of error correction and amplification at 25 °C for 60 min. By PCR identification of three DNA samples obtained through gel recycling (PCA assembly product, one round error correction amplification product, two rounds error correction amplification product), it was found that the samples after error correction could all be amplified to obtain bands of expected sizes (taking JA as an example, Supplementary Material S2). Further sequencing alignment results showed that out of the 96 single clone samples picked from JA, JB, and JC segment PCA assembly products, 4, 2, and 6 samples, respectively, were sequenced completely correctly, with an accuracy rate of 4.2 ± 2.1%; out of the 96 single clone samples picked from the products after one round of error correction, 4, 3, and 5 samples, respectively, were sequenced completely correctly, with an accuracy rate of 4.2 ± 1.0%; while out of the 96 single clone samples picked from the products after two rounds of error correction, 7, 10, and 14 samples, respectively, were sequenced completely correctly, with an increased accuracy rate of 10.7 ± 3.7% (Figure 1).

### 2.2. Optimization and Efficiency Analysis of Two-Step Error Correction Process for PCA Assembly Products

#### 2.2.1. Analysis of Correction Efficiency with Annealing Endpoint Temperature at 4 °C

**Figure 2:**
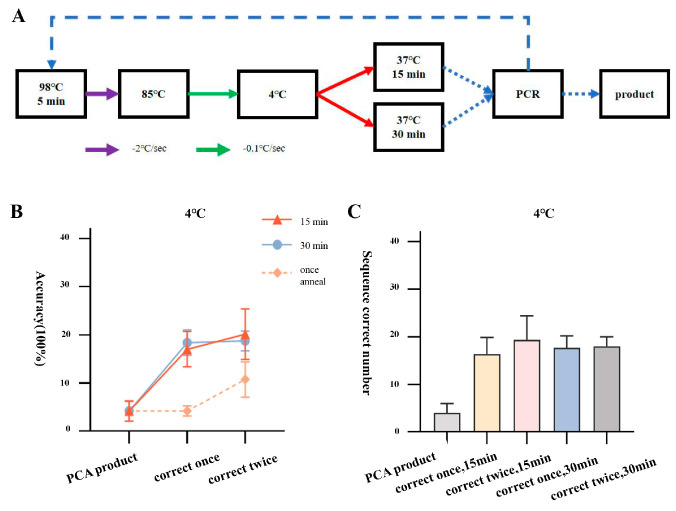
Correction process and efficiency statistics of two-step annealing to 4 °C. (**A**) Flowchart of correction process for two-step annealing to 4 °C. (**B**) Line graph showing the accuracy of amplified products after two rounds of correction with PCA assembly products and two-step annealing to 4 °C. (**C**) Number of positive samples of amplified products after two rounds of correction with PCA assembly products and two-step annealing to 4 °C.

In order to further improve the error correction efficiency, we used the same batch of PCA assembly products (accuracy of 4.2 ± 2.1%) as a template, with an annealing endpoint temperature of 4 °C, and performed two rounds of error correction and amplification using the two-step annealing error correction method (95 °C–85 °C, 85 °C–4 °C). Additionally, considering that the error correction cutting of the T7 endonuclease needs to be performed at 37 °C, we adjusted the incubation conditions of the error correction process from 25 °C for 60 min to 37 °C for 15 min or 30 min. The error-corrected products were ligated to the T vector for bacterial liquid PCR and sequencing identification. The results showed that after one round of error correction, incubation for 15 min, and transformation of 96 single clone samples picked from JA, JB, and JC fragments, there were 13, 16, and 20 completely correct sequencing samples, with an accuracy rate of 17.0 ± 3.7%. After two rounds of error correction, incubation for 15 min, and amplification of the products, 96 single clone samples picked from JA, JB, and JC fragments had 14, 20, and 24 completely correct sequencing samples, with an accuracy rate of 20.1 ± 5.2%. After one round of error correction and incubation for 30 min, 96 single clone samples were picked, with 15, 18, and 20 samples sequenced completely correctly, resulting in an accuracy rate of 18.4 ± 2.6%. After two rounds of error correction and incubation for 30 min, 96 single clone samples were picked, with 16, 18, and 20 samples sequenced completely correctly, resulting in an accuracy rate of 18.8 ± 2.1% (Figure 2). Compared to the one-step method with an accuracy rate of 10.7 ± 3.7%, there was a significant improvement. This result suggests that we can explore further error correction conditions and efficiency optimization based on the two-step annealing error correction method in order to obtain a better error correction path.

#### 2.2.2. Analysis of Correction Efficiency with Annealing Endpoint Temperature at 37 °C

**Figure 3:**
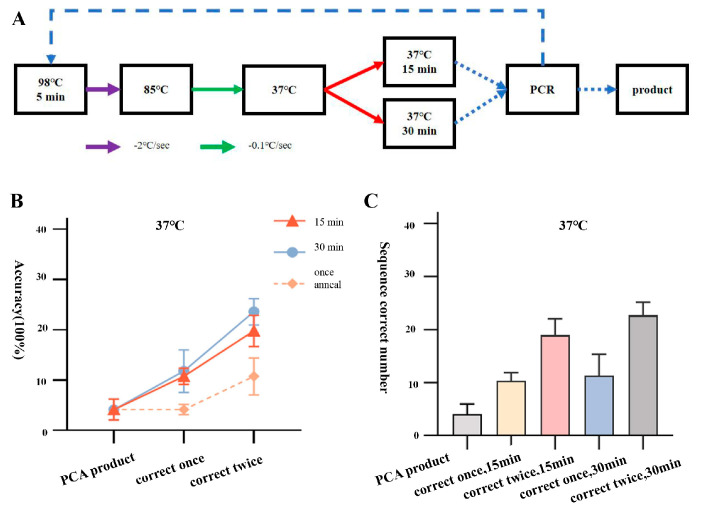
Correction process and efficiency statistics of two-step annealing to 37 °C. (**A**) Flowchart of correction process for two-step annealing to 37 °C. (**B**) Line graph showing the accuracy of amplified products after two rounds of correction with PCA assembly products and two-step annealing to 37 °C. (**C**) Number of positive samples of amplified products after two rounds of correction with PCA assembly products and two-step annealing to 37 °C.

First, we used a two-step annealing method (95 °C–85 °C, 85 °C–37 °C) with a final annealing temperature of 37 °C for the error correction cutting of T7 DNA endonuclease. We performed two rounds of error correction and amplification by enzyme cutting for 15 or 30 min each. We performed two rounds of error correction and amplification by incubating the samples for 15 or 30 min. The error-corrected products were then ligated to T vectors for bacterial PCR and sequencing identification. The results showed that after one round of error correction and 15 min of incubation, out of 96 single clone samples picked for transformation, there were 9, 10, and 12 samples with completely correct sequencing for JA, JB, and JC fragments, respectively, with an accuracy rate of 10.8 ± 1.6%. After two rounds of error correction and 15 min of incubation, there were 16, 19, and 22 samples with completely correct sequencing, with an accuracy rate of 19.8 ± 3.1%. After one round of error correction and 30 min of incubation, there were 7, 15, and 12 samples with completely correct sequencing, with an accuracy rate of 11.8 ± 4.2%. After two rounds of error correction and 30 min of incubation, there were 23, 25, and 20 samples with completely correct sequencing, with an accuracy rate of 23.6 ± 2.6% (Figure 3). We did not achieve the expected research results, as the optimal error correction efficiency under this condition only increased by around 3.5% compared to 4 °C, and further exploration is needed to determine the possible reasons for this.

#### 2.2.3. Analysis of Correction Efficiency with Annealing Endpoint Temperature at 25 °C

**Figure 4:**
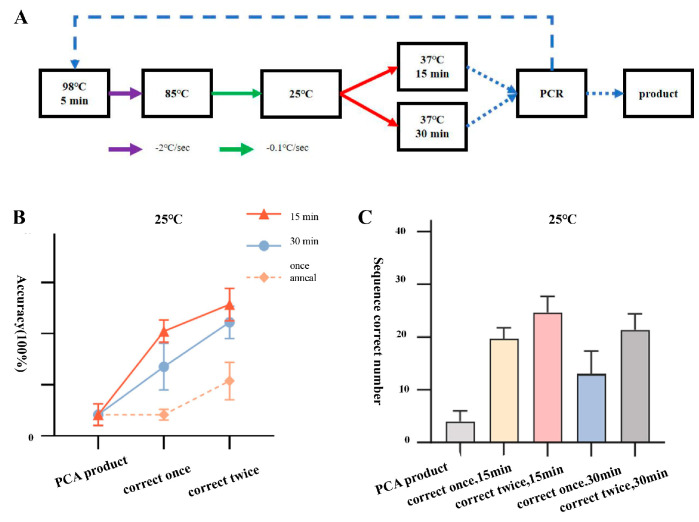
Correction process and efficiency statistics of two-step annealing to 25 °C. (**A**) Flowchart of correction process for two-step annealing to 25 °C. (**B**) Line graph showing the accuracy of amplified products after two rounds of correction with PCA assembly products and two-step annealing to 25 °C. (**C**) Number of positive samples of amplified products after two rounds of correction with PCA assembly products and two-step annealing to 25 °C.

Continuing with the same batch of PCA products as templates, we lowered the annealing endpoint temperature to 25 °C and performed two rounds of error correction and amplification using a two-step annealing method (95 °C–85 °C, 85 °C–25 °C) with enzyme digestion for 15 or 30 min. The error-corrected products were then ligated to the T vector for bacterial liquid PCR and sequencing identification. The final results showed that out of 96 single clone samples picked after transformation of JA, JB, and JC fragments following one round of error correction and 15 min of incubation, 19, 22, and 18 samples, respectively, were sequenced completely correctly, with an accuracy rate of 20.5 ± 2.2%. After two rounds of error correction and 15 min of incubation, 22, 28, and 24 samples, respectively, were sequenced completely correctly, with an accuracy rate of 25.7 ± 3.2%. For the products transformed after one round of error correction and 30 min of incubation, only 10, 11, and 18 samples out of 96 single clone samples were sequenced completely correctly, with an accuracy rate of only 13.5 ± 4.5%. After two rounds of error correction and 30 min of incubation, 18, 22, and 24 samples, respectively, were sequenced completely correctly out of 96 single clone samples picked from the amplified products, with an accuracy rate of only 22.2 ± 3.2% (Figure 4). The results indicate that the overall efficiency under this error correction condition did not show a significant improvement. The optimal error correction efficiency under this condition only increased by around 5.6% compared to 4 °C and only increased by around 2.1% compared to 37 °C.

#### 2.2.4. Analysis of Correction Efficiency with Annealing Endpoint Temperature at 16 °C

**Figure 5:**
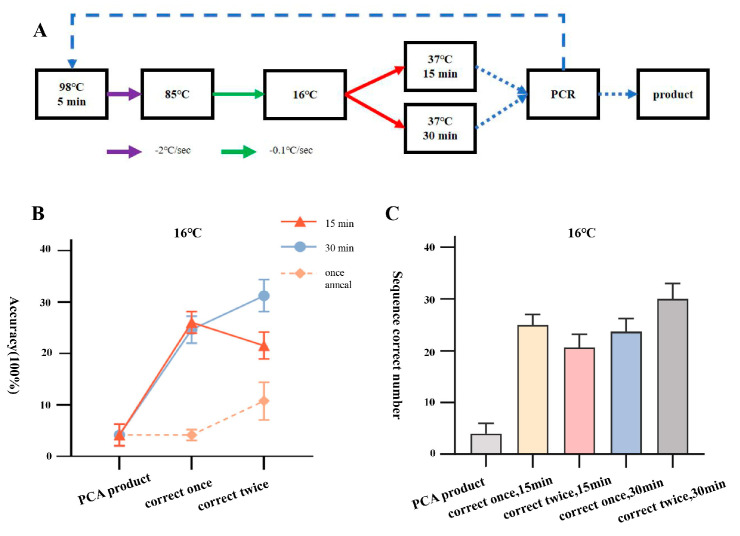
Correction process and efficiency statistics of two-step annealing to 16 °C. (**A**) Flowchart of correction process for two-step annealing to 16 °C. (**B**) Line graph showing the accuracy of amplified products after two rounds of correction with PCA assembly products and two-step annealing to 16 °C. (**C**) Number of positive samples of amplified products after two rounds of correction with PCA assembly products and two-step annealing to 16 °C.

Finally, we set the annealing endpoint temperature to 16 °C (95 °C–85 °C, 85 °C–16 °C) using the same batch of PCA products as a template and carried out two rounds of error correction and amplification. The error-corrected products were ligated to the T vector for bacterial liquid PCR and sequencing identification. The final results showed that after one round of error correction and incubation for 15 min, out of 96 single clone samples picked, there were 27, 25, and 23 samples with completely correct sequencing for JA, JB, and JC fragments, respectively, with an accuracy rate of 26.0 ± 2.1%. After two rounds of error correction and incubation for 15 min, out of 96 single clone samples picked from the amplified products, there were 18, 21, and 23 samples with completely correct sequencing for JA, JB, and JC fragments, respectively, with an accuracy rate of 21.5 ± 2.7%. After one round of error correction and incubation for 30 min, out of 96 single clone samples picked, there were 21, 24, and 26 samples with completely correct sequencing for JA, JB, and JC fragments, respectively, with an accuracy rate of 24.7 ± 2.6%; after two rounds of error correction and incubation for 30 min, out of 96 single clone samples picked from the amplified products, there were 27, 30, and 33 samples with completely correct sequencing for JA, JB, and JC fragments, respectively, with an accuracy rate of 31.3 ± 3.1% (Figure 5). We were pleasantly surprised to find that all four sets of data reached a stable level of over 20% under the annealing temperature conditions, with the optimal error correction efficiency reaching 31.3 ± 3.1%, an increase of around 11.2% compared to 4 °C. Furthermore, only one round of error correction is needed to achieve the same effect as two rounds under the other conditions.

## 3. Discussion

The rampant spread of viruses has brought profound impacts to human society, and delving into the nature and transmission mechanisms of viruses is crucial for effectively controlling epidemics and ensuring public health safety [28]. Virus engineering is one of the key technologies for exploring the essence and characteristics of viruses [29]. With the development of synthetic virus genomes, virus engineering has ushered in a new era. Synthetic virus genomes can precisely construct and manipulate the genetic material of viruses, helping to rapidly reveal the replication mechanisms, gene functions, and interactions between viruses and host cells, thereby providing new strategies and insights for the prevention, diagnosis, and treatment of viral diseases. The first virus assembled through the synthesis of oligonucleotides was the poliovirus [30], followed closely by the phiX174 bacteriophage [31]. This not only demonstrates the enhanced ability of humans to manipulate the microscopic world but also lays the foundation for the synthesis of more complex organisms in the future, marking an important milestone in virus synthesis technology. With the continuous advancement of technology, in 2008 and 2010, the Craig Venter Institute successively announced two major achievements: the successful synthesis of the genomes of two mycoplasmas [32,33]. These two feats not only pushed the boundaries of gene synthesis technology to new heights but also heralded endless possibilities in areas such as disease treatment and bioenergy in the future. After the outbreak of the COVID-19 pandemic, scientists quickly immersed themselves in the research of the novel coronavirus. A research team in Switzerland used reverse genetics to rapidly construct a live novel coronavirus in yeast based on the known genetic sequence of the virus. This profoundly demonstrates the valuable and far-reaching significance of virus genome synthesis in virus engineering and related research fields. It not only reveals the intricacy and potential of genetic manipulation but also opens up new research paths and application prospects in multiple cutting-edge technology fields such as virology, gene therapy, and vaccine development. By precisely controlling the combination of virus genes, scientists can gain a deeper understanding of the virus’s lifecycle and pathogenic mechanisms and design more effective and safe prevention and treatment strategies. This achievement is undoubtedly a major contribution to the field of virus engineering, heralding more powerful technological weapons in addressing global health challenges in the future.

The synthesis of kb-length DNA fragments is the basis for the synthesis of long viral fragments and even whole genomes. In recent years, although oligonucleotide synthesis technology has made significant progress, the synthesis of DNA sequences over 200 bp still faces challenges and relies on DNA assembly technology to achieve. There are currently various methods widely used for assembling synthetic DNA fragments, such as the overlap extension polymerase chain reaction (OE-PCR), which can synthesize genes up to 1 kb in length [34], and the two-step PCR-based DNA synthesis (PTDS) method for synthesizing long DNA fragments [35]. In addition, polymerase cycling assembly (PCA), as a key technology in the field of DNA assembly, occupies an indispensable position in gene synthesis, gene editing, synthetic biology, and other fields due to its simplicity, cost-effectiveness, and traceless products. PCA technology cleverly assembles multiple oligonucleotide fragments in the predetermined order under the catalysis of DNA polymerase by designing specific primers and templates, thereby constructing the complete DNA sequence accurately. This process, compared to traditional methods such as asymmetric extension, PTDS, etc., can easily achieve the synthesis of DNA sequences of more than 1 kb while simplifying the cumbersome steps of two-step PCR synthesis of long fragments and can directly achieve the synthesis of long sequences in one step. However, this process is not flawless, as various factors, such as the rationality of primer design, the catalytic efficiency of polymerase, and the purity and quality of the template, may introduce errors such as base mismatches, insertions, or deletions during the PCA assembly process. These errors not only directly weaken the accuracy of DNA fragments but may also add unnecessary complexity and cost to subsequent experimental steps.

Given this, current research is actively working to improve the error correction efficiency of PCA assembly DNA technology in order to reduce or eliminate these errors fundamentally. Optimizing the process parameters for error correction of the products after PCA assembly and increasing the accuracy of the products is an effective method to improve the accuracy of DNA synthesis. This study focuses on optimizing the error correction efficiency of polymerase cycling assembly (PCA) technology, which is an important step in the field of virus synthesis and is of significant research significance. Firstly, this study significantly improved the synthesis accuracy of kb-level DNA fragments by improving the error correction process of PCA technology, providing a new solution for the synthesis of long DNA fragments. Secondly, this study provides strong technical support for virus research and vaccine development. The genome of a virus is key to studying its biological characteristics and developing effective vaccines. PCA technology has broad application prospects. Rapidly synthesizing accurate viral genomes is crucial for developing new vaccines. By precisely controlling DNA sequences, efficient and safe vaccines targeting specific pathogens can be designed. This technology also helps to accelerate the vaccine development process and improve vaccine effectiveness and safety. In addition to vaccine development, DNA synthesis technology has broad application prospects in gene therapy, gene editing, biosensors, and other fields. Of course, when designing and synthesizing viruses and other laboratory materials with biosecurity risks using this technology, approval from relevant agencies must be obtained, and strict adherence to biosecurity risk procedures must be followed. When synthesizing long DNA sequences, potential biological activities, including potential pathogenicity, must be considered. It is necessary to rigorously test and validate the synthesized DNA sequences to ensure their safety and effectiveness.

In conclusion, this study has made a significant breakthrough in the field of viral engineering by optimizing the error correction efficiency of PCA technology. Its research significance is not only reflected in the advancement of technology but also in providing strong support for the development of various fields, such as virus prevention and control and biopharmaceutical design. In addition, there are still many error correction methods worth learning from and applying, including but not limited to using specific enzyme systems such as CorrectASE [27] to identify and correct erroneous base pairing, designing intelligent algorithms such as medaka [36], pilon [37], racon [38], etc., and combining high-throughput sequencing technologies to predict and correct potential errors in sequences. The design of appropriate primer sequences is also a key step in improving the final yield of correct products. In addition to the DNA works used in this study, there are many other methods worth considering, such as the thermodynamically balanced inside-out (TBIO) method for primer design [39]. In the future, with further improvements in abilities such as oligonucleotide purification, oligonucleotide synthesis, and polymerase fidelity, it is believed that the accuracy of PCA assembly products will correspondingly increase, gradually pushing PCA technology towards higher precision and efficiency, thereby providing more solid technical support for research in cutting-edge fields such as virology and synthetic biology.

Finally, this study still has some limitations, which also provide guidance for our future experiments. Firstly, the optimization and adjustment of PCA assembly were only conducted at a level of around 1 kb. Although our proposed solution can effectively correct PCA products at this level, it has only been validated at the 1 kb level. Existing literature has reported that PCA assembly can achieve assembly at several kb levels, which means that further verification is needed to determine whether this solution can stably correct errors within the limits of PCA assembly. Additionally, the final accuracy of PCA assembly products is influenced by various factors, and optimizing the correction solution is just one effective means of improving accuracy. To achieve efficient and error-free splicing, multiple systematic optimizations and a large number of orthogonal experimental screenings are still required. These will be the problems and important research subjects that we will attempt to address in the future.

## 4. Materials and Methods

### 4.1. Primer Design and Synthesis

Randomly select three 1.3 kb small DNA fragments of Japanese encephalitis virus vaccine strain (SA14-14-2) (named JA, JB, JC), and use the online software DNA Works (V.3.2.4) (https://hpcwebapps.cit.nih.gov/ (accessed on 24 October 2024)) to split them into 32–36 oligonucleotide sequences based on the principles of Tm value 62 °C, sequence length 59 bp, and Escherichia coli as the standard organism (taking JA as an example, the sequences are shown in Supplementary Material S1). These sequences were synthesized by Beijing Qiangke Biotechnology Co., Ltd. (Beijing, China) in the same batch. Add an appropriate amount of Nuclease-free water to each primer powder to make the concentration of each primer 100 µM. Then, take equal amounts of each primer into an EP tube, mix well to form a Primer Mix, and dilute with ultrapure water to a final concentration of 532 nM.

### 4.2. PCA Product Assembly

Prepare the first round PCR reaction system (9 µL Nuclease-free water, 4 µL 5×PrimeSTAR Buffer, 1.6 µL dNTP, 5 µL Primer Mix, 0.4 µL PrimeSTAR HS DNA Polymerase) using the high-fidelity polymerase PrimeSTAR HS (TaKaRa, R010A). Run the first round of PCR reaction (pre-denaturation at 95 °C for 3 min followed by denaturation at 95 °C for 15 s, annealing at 55 °C for 30 s, extension at 72 °C for 80 s for a total of 15 cycles; then, final extension at 72 °C for 10 min; finally, store at 10 °C). Then, using the first round PCR product as a template, set up the second round PCR reaction system with the 5′ primer and 3′ primer (31.5 µL Nuclease-free water, 10 µL 5×PrimeSTAR Buffer, 4 µL dNTP, 2.5 µL first round PCR product, 1 µL 5′ primer, 1 µL 3′ primer, 1 µL PrimeSTAR HS DNA Polymerase). Run the second round of PCR reaction (pre-denaturation at 95 °C for 3 min followed by denaturation at 95 °C for 15 s, annealing at 55 °C for 15 s, extension at 72 °C for 80 s for a total of 15 cycles; then, final extension at 72 °C for 10 min; finally, store at 10 °C). After amplification, the PCR products are identified using 1% agarose gel electrophoresis, and the gel-purified products from the second round of PCR are then assembled to form the PCA assembly product.

### 4.3. PCA Product Error Correction

Use an appropriate amount of PCA product as a template, set up error correction reaction system with NEB buffer 2 (1 µL NEB buffer 2, 25 ng/µL PCA assembly product, add nuclease-free water to a total volume of 15 µL), mix thoroughly and run the reaction in a thermal cycler (heat to 95 °C for 2 min, then cool to 4 °C within 17 min). Then add 1 µL of T7 Endonuclease I (NEB, M0302L), mix well, and incubate at 25 °C in a thermal cycler for 60 min. Heat at 65 °C for 20 min to deactivate the enzyme activity, which is the first round of error correction product.

Using the template of the first round of error correction products, set up the amplification reaction system (11.6 µL Nuclease-free water, 4 µL 5 × PrimeSTAR Buffer, 1.6 µL dNTP, 0.4 µL PrimeSTAR HS DNA Polymerase, 0.2 µL 5′ primer, 0.2 µL 3′ primer, 2 µL of the first round of error correction product). Run the first round of error correction product amplification reaction (pre-denaturation at 95 °C for 3 min followed by denaturation at 95 °C for 15 s, annealing at 55 °C for 15 s, extension at 72 °C for 80 s for a total of 15 cycles; then, final extension at 72 °C for 10 min; finally, store at 10 °C). After amplification, use 1% agarose gel electrophoresis for identification and recover PCR products from the gel, which are the amplified products after the first round of error correction.

Using the first round error correction and amplification product as a template, perform the second round error correction and amplification reaction under the same conditions. After completion, identify the error correction products of the second round by 1% agarose gel electrophoresis, gel recovery PCR product, which is the PCA assembly of the second round error correction product.

### 4.4. PCA Product Error Correction Optimization Process

Utilize the PCA products prepared in the same batch to set up an error correction system according to the above conditions (Section 4.3). After mixing, heat at 95 °C for 5 min in a thermal cycler, then perform a two-step cooling reaction: first, rapidly cool from 95 °C to 85 °C (−2 °C/s); then, slowly cool from 85 °C to the endpoint temperature of 4 °C, 37 °C, 25 °C, or 16 °C (−0.1 °C/s). After reaching the set endpoint temperature, add 1 µL of T7 Endonuclease I to 9 µL of the reaction product, mix, and then heat at 37 °C in the thermal cycler for 15 min or 30 min. Finally, heat at 65 °C for 20 min to deactivate the enzyme activity, which is the first round of the two-step correction product.

Using the first-round two-step error correction product as a template with different endpoint temperatures, set up the first-round error correction product amplification reaction system (11.6 µL Nuclease-free water, 4 µL 5 × PrimeSTAR Buffer, 1.6 µL dNTP, 0.4 µL PrimeSTAR HS DNA Polymerase, 0.2 µL 5′ end primer, 0.2 µL 3′ end primer, 2 µL first-round error correction product). Run the first-round two-step error correction product amplification reaction (pre-denaturation at 95 °C for 3 min; then denaturation at 95 °C for 15 s, annealing at 55 °C for 15 s, extension at 72 °C for 80 s for a total of 15 cycles followed by a final extension at 72 °C for 10 min; finally, store at 10 °C). After amplification, use 1% agarose gel electrophoresis for identification, recover PCR products from the gel, and obtain the amplified product after the first-round two-step error correction.

Using the first-round two-step error correction and amplification product as a template, perform the second-round two-step error correction and amplification reaction under the same conditions. After completion, identify the error correction products of the second round by 1% agarose gel electrophoresis, gel recovery PCR product, which is the second-round two-step error correction product.

### 4.5. Efficiency Assessment

Mix the PCA-assembled error-corrected final products of each group with the pJET1.2/Blunt Cloning Vector vector in a 1:1 ratio by mass and incubate at 22 °C for 30 min. The transformation product is transferred to chemically competent cells of Escherichia coli DH10B: incubate on ice for 30 min, heat shock at 42 °C for 90 s, and then leave on ice for 2 min. Add 700 µL of antibiotic-free LB medium to the mixture and culture at 37 °C with shaking at 150 rpm for 1 h. Spread the transformation product onto Amp-LB solid plates and invert for cultivation at 37 °C. Pick single colonies, shake at 37 °C and 200 rpm for 6 h, then take an appropriate amount of bacterial liquid as a template for PCR reaction using the 5′ primer and 3′ primer from the Primer Mix (0.5 µL 5′ primer, 0.5 µL 3′ primer, 1 µL bacterial template, 12.5 µL Hot Start Taq 2 × Master Mix, 10.5 µL Nuclease-free water). Run the amplification reaction (pre-denaturation at 95 °C for 30 s followed by denaturation at 95 °C for 30 s, annealing at 55 °C for 1 min, extension at 68 °C for 1 min for a total of 30 cycles; then, final extension at 68 °C for 5 min; finally, store at 4 °C). After amplification, the products were identified by 1% agarose gel electrophoresis, and the correctly sized fragments were recovered and sent to Sangon Biotech (Shanghai) Co., Ltd. for sequencing. To calculate the assembly or correction efficiency, divide the number of samples with correct sequencing alignment by the total number of sequenced samples and multiply by 100%.

## 5. Conclusions

**Figure 6:**
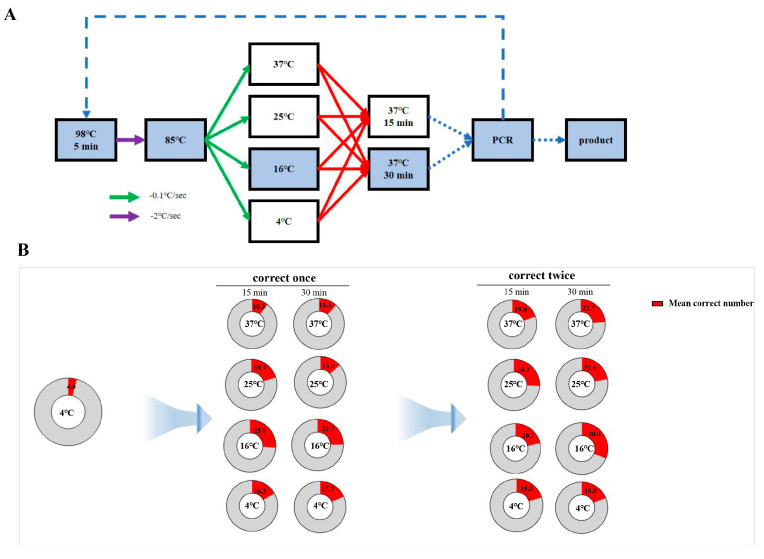
Correction process and efficiency statistics of two-step annealing to 37/25/16/4 °C. (**A**) Flowchart of correction process for two-step annealing to 37/25/16/4 °C. (**B**) Number of positive samples of amplified products after two rounds of correction with PCA assembly products and two-step annealing to 37/25/16/4 °C.

We used the same batch of around 1.3 kb PCA products as a template and attempted a total of 16 error correction methods (Supplementary Material S3). By adjusting the error correction from one-step annealing to two-step annealing and optimizing the annealing endpoint temperature from 4 °C to 16 °C, the final accuracy of the products increased from 26.0 ± 2.1% after two rounds of correction to achieving the same level with just one round of correction. Meanwhile, the final accuracy improved from 4.2 ± 2.1% without correction to 31.3 ± 3.1% after two rounds of correction (Figure 6, Supplementary Material S4). Among all error correction results, we ultimately established that using 16 °C as the final annealing temperature and 30 min as the final incubation time for two rounds of correction significantly increased the correction efficiency.

This study not only confirmed the feasibility of using T7 endonuclease for error correction in PCA assembly products but also filled the data gap for error correction of kb-scale DNA fragments. It also provided experimental parameters and standardized experimental procedures for DNA fragment assembly under limited conditions, making it easier for future researchers to efficiently utilize this method for DNA assembly, thus supporting subsequent life science experimental techniques.

## Data Availability

Data is contained within the article or Supplementary Material.

## Supplementary Materials

The following supporting information can be downloaded at: https://www.mdpi.com/article/10.3390/ijms252111514/s1.
