# Supplementary material for: Optimization of PCA Error Correction Conditions to Improve Efficiency of Virus Genome De Novo Synthesis
Source: Int J Mol Sci. 2024 Oct 26;25(21):11514. doi: 10.3390/ijms252111514 (PMC11547124; doi:10.3390/ijms252111514)
Supplement: Supplementary file 1 [file ijms-25-11514-s001.zip › ijms-3209731-supplementary.pdf]

Supplementary Material S1

JA primer sequence

Primer name	Primer sequence (5′—3′)	bp
A1（5′）	GGATCCGAATAAGGGCGACACGGAAATGTCACCCAACTG	39
A2	ATAATATGGCTAATGGCCAATATTGAAGATGCTGAAGATCAGT TGGGTGACATTTCCGT	59
A3	CAATATTGGCCATTAGCCATATTATTCATTGGTTATATAGCATA AATCAATATTGGCTA	59
A4	TGATATAGATACAACGTATGCAATGGCCAATAGCCAATATTGA TTTATGCTATATAACC	59
A5	CCATTGCATACGTTGTATCTATATCATAATATGTACATTTATAT TGGCTCATGTCCAAT	59
A6	ACTAGTCAATAATCAATGCCAACATGGCGGTCATATTGGACAT GAGCCAATATAAATGT	59
A7	ATGTTGGCATTGATTATTGACTAGTTATTAATAGTAATCAATTA CGGGGTCATTAGTTC	59
A8	AGTTATGTAACGCGGAACTCCATATATGGGCTATGAACTAATG ACCCCGTAATTGATTA	59
A9	GGAGTTCCGCGTTACATAACTTACGGTAAATGGCCCGCCTGGC TGACCGCCCAACGACC	59
A10	GTTACTATGGGAACATACGTCATTATTGACGTCAATGGGCGGG GGTCGTTGGGCGGTCA	59
A11	TAATGACGTATGTTCCCATAGTAACGCCAATAGGGACTTTCCA TTGACGTCAATGGGTG	59
A12	TTGATGTACTGCCAAGTGGGCAGTTTACCGTAAATACTCCACC CATTGACGTCAATGGA	59
A13	CCCACTTGGCAGTACATCAAGTGTATCATATGCCAAGTCCGCC CCCTATTGACGTCAAT	59
A14	TCATGTACTGGGCATAATGCCAGGCGGGCCATTTACCGTCATT GACGTCAATAGGGGGC	59
A15	GGCATTATGCCCAGTACATGACCTTACGGGACTTTCCTACTTG GCAGTACATCTACGTA	59
A16	CCAAAACCGCATCACCATGGTAATAGCGATGACTAATACGTA GATGTACTGCCAAGTAG	59
A17	CATGGTGATGCGGTTTTGGCAGTACACCAATGGGCGTGGATAG CGGTTTGACTCACGGG	59

A18	ACAAACTCCCATTGACGTCAATGGGGTGGAGACTTGGAAATC<br>CCCGTGAGTCAAACCGC	59
A19	TTGACGTCAATGGGAGTTTGTTTTGGCACCAAAATCAACGGGA<br>CTTTCCAAAATGTCGT	59
A20	ACGCCTACCGCCCATTTGCGTCAACGGGGCGGGGTTATTACGA<br>CATTTTGGAAAGTCCC	59
A21	AAATGGGCGGTAGGCGTGTACGGTGGGAGGTCTATATAAGCA<br>GAGCTCGTTTAGTGAAC	59
A22	GATACTAAGCCAAGAAGTTCACACGATTAAACTTCTCGGTTCA<br>CTAAACGAGCTCTGCT	59
A23	GTGTGAACTTCTTGGCTTAGTATCGTTGAGAAGAATCGAGAGA<br>TTAGTGCAGTTTAAAC	59
A24	TTTAGTCATGGTTGTTCTTCCGTTCTAAAAAACTGTTTAAACTG<br>CACTAATCTCTCGAT	59
A25	CGGAAGAACAACCATGACTAAAAAACCAGGAGGGCCCGGGA<br>AAAACCGGGCCATCAATA	59
A26	CCACTAGTGGGAATACGCGGGGTAATCCGCGTTTCAGCATATT<br>GATGGCCCGGTTTTTC	59
A27	CGCGTATTCCCACTAGTGGGAGTGAAGAGGGTAGTGATGAGCT<br>TGTTGGACGGCAGAGG	59
A28	ACTTGAAGAACGTGATAAGAGCCAGCACAAATCGTACTGGCC<br>CTCTGCCGTCCAACAAG	59
A29	GCTCTTATCACGTTCTTCAAGTTCACAGCATTAGCCCCGACCA<br>AGGCTCTTTTGGGCCG	59
A30	TAAGATGTTTCATTGCCACACTCTTCTCCACTGCTCTCCATCGG<br>CCCAAAAGAGCCTTG	59
A31	GAGTGTGGCAATGAAACATCTTACCAGTTTCAAACGAGAACTT<br>GGAACACTCATCGACG	59
A32	TCCTCTTTTGTTTTGTTTCTTGCCCCGTTTATTCACGGCGTCGAT<br>GAGTGTTCCAAGTT	59
A33	GCAAGAAACAAAACAAAAGAGGAGGGAATGAGAGCTCGATC<br>ATGTGGCTTGCCAGCTTG	59
A34（3′）	GAATTCCGATTGCCAAGCTGGCAAGCCACA	30

JB primer sequence

Primer name	Primer sequence (5′—3′)	bp
B1（5′）	TTGAACTCTCCTACTCTGGGAGTGATGGCCCCTG	34
B2	TCATGTCATTGAGGCTCGCAACGGAAACAATCGGAATTTTGCA GGGGCCATCACTCCCA	59
B3	TTGCGAGCCTCAATGACATGACCCCCGTTGGGCGGCTGGTGAC AGTGAACCCCTTCGTC	59
B4	ATCTCGACCAGCACCTTTGAGTTGGCACTGGAAGTCGCGACGA AGGGGTTCACTGTCAC	59
B5	CTCAAAGGTGCTGGTCGAGATGGAACCCCCCTTCGGAGACTCC TACATCGTAGTTGGAA	59
B6	TTTGTGCCAATGGTGGTTGATCTGCTTGTCTCCCCTTCCAACTA CGATGTAGGAGTCTC	59
B7	GATCAACCACCATTGGCACAAAGCTGGAAGCACGCTGGGCAA GGCCTTTTCAACAACTT	59
B8	GTGTCGCCCAACGCTGCCAGTCTTTGAGCTCCCTTCAAAGTTGT TGAAAAGGCCTTGCC	59
B9	GCAGCGTTGGGCGACACAGCCTGGGACTTTGGCTCTATTGGAG GGGTCTTCAACTCCAT	59
B10	TGAAGGCACCACCAAACACTTGGTGAACGGCTCTTCCTATGGA GTTGAAGACCCCTCCA	59
B11	AAGTGTTTGGTGGTGCCTTCAGAACACTCTTTGGGGGAATGTCT TGGATCACACAAGGG	59
B12	GTTGACGCCCATCCAGAGCAGTAGGGCACCCATTAGCCCTTGT GTGATCCAAGACATTC	59
B13	GCTCTGGATGGGCGTCAACGCACGAGACCGATCAATTGCTTTG GCCTTCTTAGCCACAG	59
B14	GCATGCACATTGGTCGCTAAGAACACGAGCACACCTCCTGTGG CTAAGAAGGCCAAAGC	59
B15	CTTAGCGACCAATGTGCATGCTGACACTGGATGTGCCATTGAC ATCACAAGAAAAGAGA	59

B16	TCGTTGTGCACGAAGATGCCACTTCCACATCTCATCTCTTTTCT TGTGATGTCAATGGC	59
B17	GGCATCTTCGTGCACAACGACGTGGAAGCCTGGGTGGATAGG TATAAATATTTGCCAGA	59
B18	TGTGGACGATCTTCGCTAGGGATCTGGGCGTTTCTGGCAAATA TTTATACCTATCCACC	59
B19	CCTAGCGAAGATCGTCCACAAAGCGCACAAGGAAGGCGTGTG CGGAGTCAGATCTGTCA	59
B20	GTCCCTTACGGCTTCCCACATTTGGTGCTCCAGTCTAGTGACAG ATCTGACTCCGCACA	59
B21	GTGGGAAGCCGTAAGGGACGAATTGAACGTCCTGCTCAAAGA GAATGCAGTGGACCTCA	59
B22	GAGCGATATCTTCCCACGGGCTTGTTCACAACCACACTGAGGT CCACTGCATTCTCTTT	59
B23	CCCGTGGGAAGATATCGCTCAGCCCCTAAACGCCTATCCATGA CGCAAGAGAAGTTTGA	59
B24	GAGGATGCTTTTTCCCCATGCTTTCCAGCCCATTTCAAACTTCT CTTGCGTCATGGATA	59
B25	GCATGGGGAAAAAGCATCCTCTTTGCCCCGGAATTGGCTAACT CCACATTTGTCGTAGA	59
B26	TGTGCTCATCAGGGCATTCCTTTGTCTCAGGTCCATCTACGACA AATGTGGAGTTAGCC	59
B27	GGAATGCCCTGATGAGCACAGAGCTTGGAACAGCATGCAAAT CGAAGACTTCGGCTTTG	59
B28	CTCTAATTTTCAGCCACACACGGGTTGATGTGATGCCAAAGCC GAAGTCTTCGATTTGC	59
B29	CCGTGTGTGGCTGAAAATTAGAGAGGAGAGCACTGACGAGTG TGATGGAGCGATCATAG	59
B30	CACTATGGACTGCCACATGTCCTTTGACAGCCGTGCCTATGAT CGCTCCATCACACTCG	59
B31	GGACATGTGGCAGTCCATAGTGACTTGTCGTACTGGATTGAGA GTCGCTACAACGACAC	59

B32	ATTTGACCTCTCCAAAGACTGCCCTCTCAAGTTTCCATGTGTCG TTGTAGCGACTCTCA	59
B33	GGCAGTCTTTGGAGAGGTCAAATCTTGCACTTGGCCAGAGACA CACACCCTTTGGGGAG	59
B34（3′）	CTTTCCTCAACATCATCTCCCCAAAGGGTGTGTGTC	36

JC primer sequence

Primer name	Primer sequence (5′—3′)	bp
C1（5′）	GAGGAAAGTGAACTCATCATTCCGCACACCATAGCCG	37
C2	TGTCTTATACCCTTCCCTCCGATTGTGCTTGCTTTTTGGTCCGGC TATGGTGTGCGGAA	59
C3	TCGGAGGGAAGGGTATAAGACACAAAACCAGGGACCTTGGG ATGAGAATGGCATAGTCT	59
C4	GGTGACTTTTGTCCCTGGGCAATAATCAAAGTCCAAGACTATG CCATTCTCATCCCAAG	59
C5	GCCCAGGGACAAAAGTCACCATTACAGAGGATTGTAGCAAGA GAGGCCCTTCGGTCAGA	59
C6	AGCACCAGTCAGTGATCAACTTTCCACTGTCAGTAGTGGTTCT GACCGAAGGGCCTCTC	59
C7	AAAGTTGATCACTGACTGGTGCTGTCGCAGTTGCTCCCTTCCGC CCCTACGATTCCGGA	59
C8	AACAGGTCTGATTTCCATTCCGTACCAGCAGCCATTTTCTGTCC GGAATCGTAGGGGCG	59
C9	ACGGAATGGAAATCAGACCTGTTATGCATGATGAAACAACAC TCGTCAGATCACAGGTT	59
C10	CTGAAAAGGGTCAACCATTTCACCTTTGAAAGCATGAACCTGT GATCTGACGAGTGTTG	59

C11	GGTGAAATGGTTGACCCTTTTCAGCTGGGCCTTCTGGTGATGTTTCTGGCCACCCAGGA	59
C12	AGGAATGGTCAATCTGGCCGTCCACCTCTTGCGAAGGACTTCCTGGGTGGCCAGAAACA	59
C13	CGGCCAGATTGACCATTCCTGCGGTTTTGGGGGTCCTACTTGTGCTGATGCTTGGGGGT	59
C14	CGACTAGCACCACATACCTCGCCAAATCAGTGTAAGTGATACCCCCAAGCATCAGCACA	59
C15	CGAGGTATGTGGTGCTAGTCGCTGCTGCTTTCGCAGAGGCCAACAGTGGAGGAGACGTC	59
C16	TGGTTGGATCTTAAAAACAGCAATCAAAGCAAGGTGCAGGACGTCTCCTCCACTGTTGG	59
C17	TGATTGCTGTTTTTAAGATCCAACCAGCATTTTTAGTGATGAACATGCTTAGCACGAGA	59
C18	CCCTAGGACCAGAACCACGTTTTCTTGGTTCGTCCATCTCGTGCTAAGCATGTTCATCA	59
C19	ACGTGGTTCTGGTCCTAGGGGCTGCCTTTTTCCAATTGGCCTCAGTAGATCTGCAAATA	59
C20	CTATAGCAGCGGCATTCAGGATTCCGTGGACTCCTATTTGCAGATCTACTGAGGCCAAT	59
C21	TCCTGAATGCCGCTGCTATAGCATGGATGATTGTCCGAGCGATCACCTTCCCCACAACC	59
C22	CGGAGTTAGAAGCGCTAAGACTGGCATGGTGACGGAGGAGGTTGTGGGGAAGGTGATCG	59
C23	AGTCTTAGCGCTTCTAACTCCGGGGATGAGGGCTCTATACCTAGACACTTACAGAATCA	59
C24	AGCAGGGAGCAAATCCCTATGACGAGGAGGATGATTCTGTAAGTGTCTAGGTATAGAGC	59
C25	CATAGGGATTTGCTCCCTGCTGCACGAGAGGAAAAAGACCATGGCGAAAAAGAAAGGAG	59
C26	ATCCAGTGGATGTGAGCGCTAAGCCCAAGAGTACAGCTCCTTT	59

	CTTTTTCGCCATGGTC	
C27	TAGCGCTCACATCCACTGGATGGTTCTCGCCCACCACTATAGC<br>TGCCGGACTAATGGTC	59
C28	TCAGTAGCTGGCCACCCTCTCTTCTTGTTTGGGTTGCAGACCAT<br>TAGTCCGGCAGCTAT	59
C29	GAGGGTGGCCAGCTACTGAGTTTTTGTCGGCAGTTGGATTGAT<br>GTTTGCCATCGTAGGT	59
C30	TATTGACATGGATTCAATATCCAACTCGGCCAAACCACCTACG<br>ATGGCAAACATCAATC	59
C31	CGAGTTGGATATTGAATCCATGTCAATACCCTTCATGCTGGCA<br>GGTCTCATGGCAGTGT	59
C32	TCAAGCCACATATCTGTTGCTTTTCCTGACACCACGTAGGACA<br>CTGCCATGAGACCTGC	59
C33	GAAAAGCAACAGATATGTGGCTTGAACGGGCCGCCGACATCA<br>GCTGGGATATGGGTGCT	59
C34（3’）	GCTTCCTGTGATTGCAGCACCCATATCCCAGCTGAT	36

Supplementary Material S2

Efficiency analysis of one-step annealing to 4℃. **(A).** Identification results of JA fragments amplified based on the PCA assembly product; .**(B).** Identification of positive clones of JA conversion products: PCA assembly products transformed into single clone samples in Escherichia coli for identification; **(C).** Identification of positive clones of JA conversion products: amplification products after the first round of correction transformed into single clone samples in Escherichia coli for identification; **(D).** Identification of positive clones of JA conversion products: amplification products after the second round of correction transformed into single clone samples in Escherichia coli for identification;We can preliminarily observe whether bands have been amplified through agarose gel electrophoresis, but the specific sequence correctness still needs to be further confirmed through sequencing

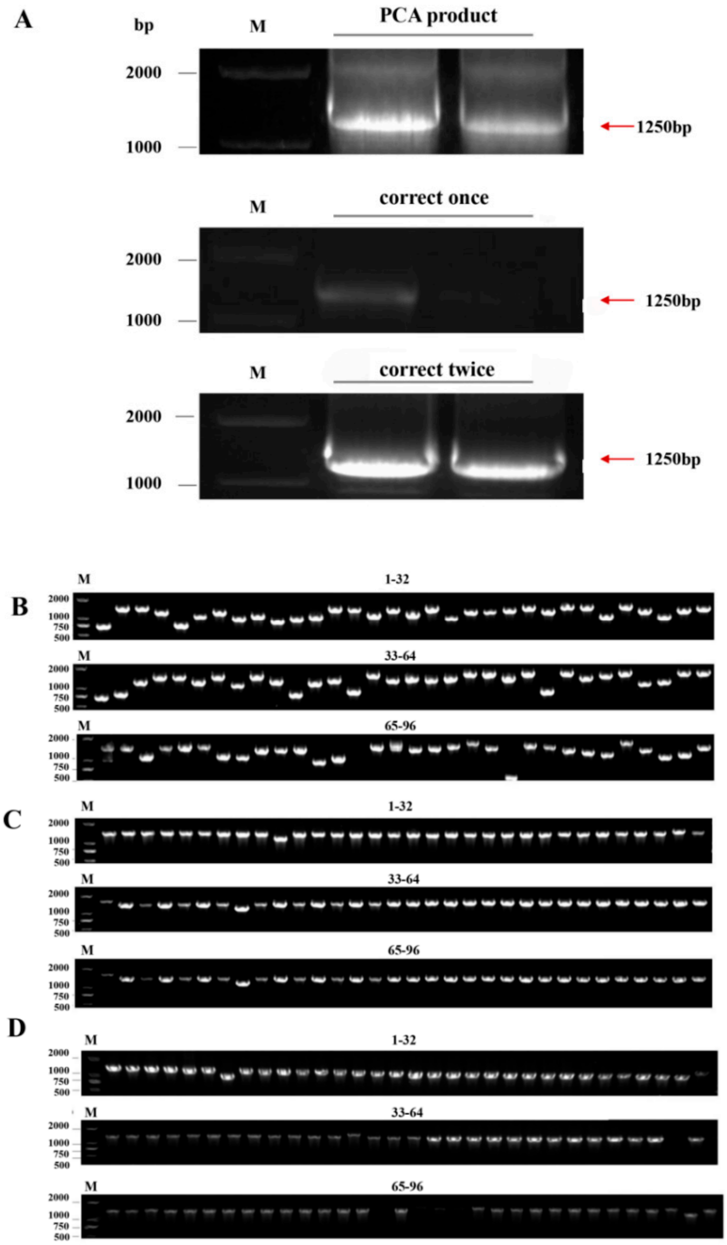

Supplementary Material S3

PCA product error correction process flowchart and comparison of differences before and after optimization. **(A).** Product error correction process and distribution of mismatch situations, red represents the wrong base; **(B)**. Comparison of Error Correction Methods Before and After Optimization.

A

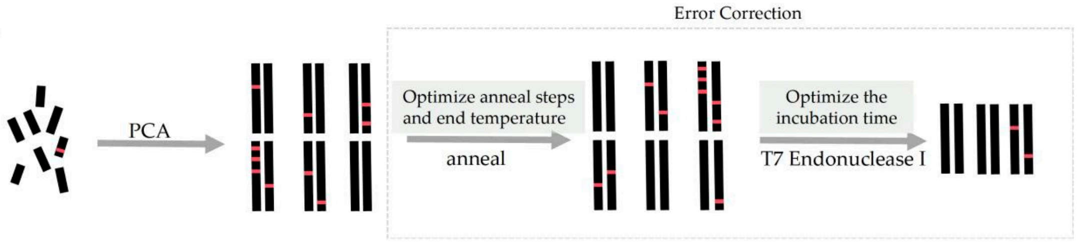

B

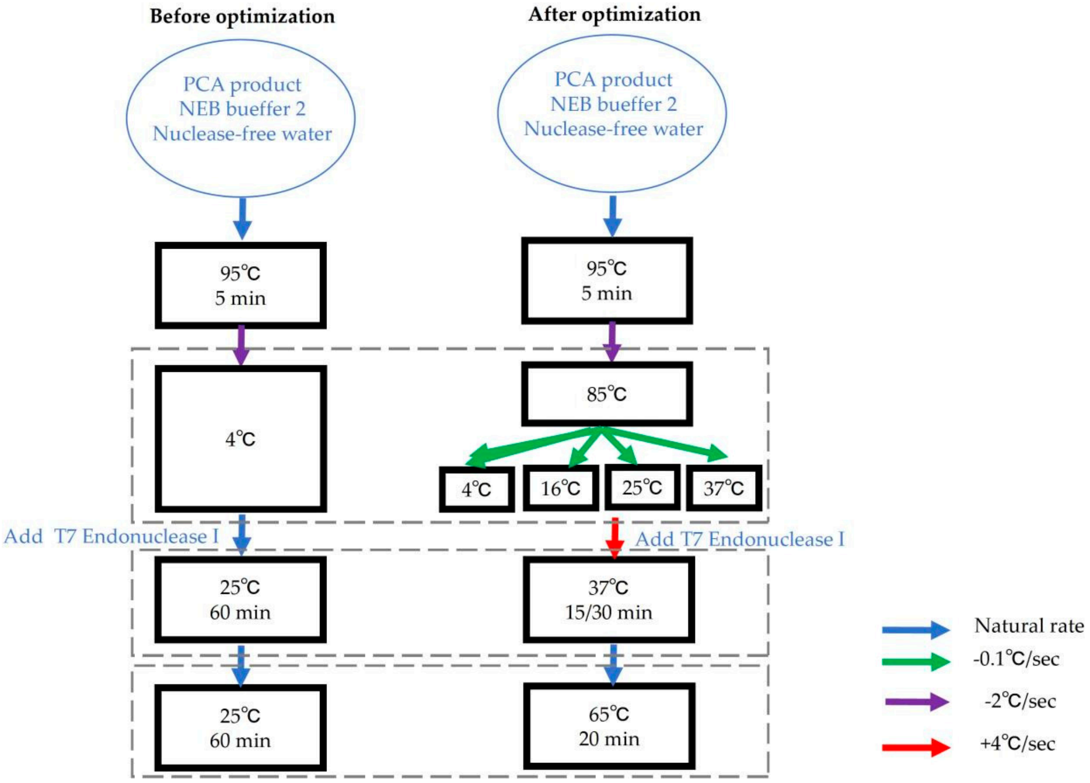

Supplementary Material S4

Error correction efficiency summary

Endpoint Temperature	Incubation conditions	Error Correction	Sequence correct number	Accuracy(100%)
		PCA assembly product	4.0±2.0	4.2±2.1%
4℃ (one-step anneal)	37℃，15 min	one round error correction	4.0±1.0	4.2±1.0%
		two rounds error correction	10.3±3.5	10.7±3.7%
37℃ (two-step anneal)	37℃，15 min	one round error correction	10.3±1.5	10.8±1.6%
		two rounds error correction	19±3	19.8%±3.1%
	37℃，30 min	one round error correction	11.3±4.0	11.8±4.2%
		two rounds error correction	22.7±2.5	23.6±2.6%
25℃ (two-step anneal)	37℃，15 min	one round error correction	19.7±2.1	20.5±2.2%
		two rounds error correction	24.7±3.1	25.7±3.2%
	37℃，30 min	one round error correction	13±4.4	13.5±4.5%
		two rounds error correction	21.3±3.1	22.2±3.2%
16℃ (two-step anneal)	37℃，15 min	one round error correction	25.0±2.0	26.0±2.1%
		two rounds error correction	20.7±2.5	21.5±2.7%
	37℃，30 min	one round error correction	23.7±2.5	24.7±2.6%
		two rounds error correction	30±3.0	31.3±3.1%
4℃ (two-step anneal)	37℃，15 min	one round error correction	16.3±3.5	17.0±3.7%
		two rounds error correction	19.3±5.0	20.1±5.2%
	37℃，30 min	one round error correction	17.7±2.5	18.4±2.6%
		two rounds error correction	18±2.0	18.8±2.1%
